# Causal Relationship of Genetically Predicted Serum Micronutrients Levels With Sarcopenia: A Mendelian Randomization Study

**DOI:** 10.3389/fnut.2022.913155

**Published:** 2022-06-22

**Authors:** Tingting Sha, Wei Li, Hongyi He, Jing Wu, Yilun Wang, Hui Li

**Affiliations:** ^1^Hunan Key Laboratory of Joint Degeneration and Injury, Changsha, China; ^2^Department of Orthopedics, Xiangya Hospital, Central South University, Changsha, China

**Keywords:** sarcopenia, Mendelian randomization, micronutrients, minerals, vitamins

## Abstract

**Objectives:**

Previous observational studies have suggested associations between concentrations of several circulating micronutrients and sarcopenia. However, the causality inferred from those studies was subjected to residual confounding and reverse causation. Therefore, we aimed to examine the causal effects of the levels of genetically predicted serum micronutrients on sarcopenia.

**Methods:**

Single nucleotide polymorphisms (SNPs) were chosen from large-scale genome-wide association studies of participants only with European descent and were used as genetic instruments for the levels of 10 serum micronutrients (calcium, magnesium, selenium, copper, iron, zinc, Vitamin A, Vitamin B12, Vitamin D, and Vitamin E). Sarcopenia was defined by referencing to the 2019 definition given by the European Working Group on Sarcopenia in Older People (EWGSOP). A two-sample Mendelian randomization (MR) analysis was carried out to examine the associations between the levels of genetically predicted serum micronutrients and the risk of sarcopenia. Then, sensitivity analyses (including weighted median, MR-Egger and leave-one-out sensitivity analyses) were performed to evaluate the robustness of study findings. The estimates were presented as odds ratio (OR) with their 95% confidence intervals (CIs) per one standard deviation (SD) increase in the exposures.

**Results:**

A total of 378,635 UK Biobank participants, including 572 participants who were identified with sarcopenia, were included in this study. The iron status was shown to have a clear effect on the risk of sarcopenia based on MR analyses. The per one SD increment in the genetically-determined serum iron level corresponded to a 53% increase in the risk of sarcopenia (OR = 1.53, 95% CI: 1.31–1.78, *P* = 0.001). The exclusion of SNPs of the circulating iron level (i.e., rs1799945 SNP, rs1800562 SNP or rs855791 SNP) did not attenuate the magnitude of the signal in MR analysis. There was little evidence supporting the associations between other remaining micronutrients and sarcopenia.

**Conclusions:**

An increased risk of sarcopenia was observed with a genetically higher concentration of iron, suggesting that iron may play a role in the occurrence or development of sarcopenia.

## Introduction

Sarcopenia is a progressive and generalized skeletal muscle disorder accompanied by accelerated loss of muscle mass and degradation of function, which is found to have high morbidity and mortality (1, 2). The prevalence of sarcopenia in the elderly population (age >60 years) was reported to be around 5–10% (3, 4). With the aging of the world's population, sarcopenia is likely to impose an increasing burden on public health resources in the future (5). However, the risk factors of sarcopenia remain insufficiently delineated, especially for those nutritional factors drawing considerable attention as modifiable risk factors and potentially therapeutic targets (6).

Micronutrients are critical for physiological functions of human being, and recent studies have found potential associations between micronutrients and muscle performance in elderly individuals (7, 8). The associations between disturbances of several serum micronutrients (calcium, magnesium, selenium, copper, iron, zinc, Vitamin A, Vitamin B12, Vitamin D, and Vitamin E) and sarcopenia have been identified in previous observational studies, but the findings are controversial (6, 9–16). Besides, given the observational design of these studies, the causal relationships between the levels of aforementioned serum micronutrients and the risk of sarcopenia remained unknown to a large extent. The existence of a potential causal association between a risk factor of interest and a target disease can be assessed by the Mendelian randomization (MR) approach using genetic variants as instrumental variables (17–19). As the genotype is allocated randomly at conception, genetic variants are not influenced by potential confounding factors such as environmental exposures, and cannot be altered by the occurrence of a disease (20). Nevertheless, to our best knowledge, the effects of micronutrients in sarcopenia have not been clearly elucidated on a large scale with the use of MR.

In this study, a two-sample MR analysis was carried out to explore the potential causal associations between the levels of serum micronutrients (calcium, magnesium, selenium, copper, iron, zinc, Vitamin A, Vitamin B12, Vitamin D, and Vitamin E) and the risk of sarcopenia. Our findings will help identify the mechanisms how micronutrients act as risk factors for sarcopenia and provide potential implications in the prognosis and treatment of sarcopenia.

## Methods

### Population

The UK Biobank is a prospective cohort study containing data of about 500,000 participants aged from 40 to 69 years who are recruited from the UK National Health Services between 2006 and 2010. Its study protocol was available online and more details can be found from the literature (21). To minimize potential confounding caused by ancestry, our study was restricted to the participants of white European descent only, and accordingly, the UK Biobank dataset was quality-controlled and filtered by removing individuals with non-white European ancestry. Other exclusion criteria were sex mismatch, excess heterozygosity, data deficiency (i.e., with missing information on grip strength, standing height, and fat-free mass), and second-degree relatives (22, 23). This study has been conducted using the UK Biobank Resource under Application Number 77,646.

### Defining Genetic Instruments

Single nucleotide polymorphisms (SNPs) were derived from the recently-conducted large-scale, European ancestry genome-wide association studies (GWAS) and were used as variables of genetic instruments for serum micronutrients (calcium, magnesium, selenium, copper, iron, zinc, Vitamin A, Vitamin B12, Vitamin D and Vitamin E). All SNPs associated with a corresponding exposure at the genome-wide significance level (*P* <5 ×10^−8^) were extracted. From all genetic instruments, three independent ones were selected for systemic iron status, which could explain ~3.8% of the variation in blood iron (24). Six calcium- (25) or magnesium-SNPs (26) were identified by published GWAS studies, which could explain 0.37 and 1.62% of the variance in blood calcium or magnesium levels, respectively. Two significant SNPs associated with the blood copper level were selected, accounting for 5% of the phenotypic variance of the copper level (27). Similarly, two SNPs were selected as instrumental variables for zinc, explaining 4.59% of the variance of its concentration (27). Two vitamin A- (28) or vitamin E-SNPs (29) were identified according to published GWAS studies, which could explain 2.3 and 1.7% of the variance in blood vitamin A or vitamin E levels, respectively. Eight independent SNPs were selected as instrumental variables for circulating vitamin B12 that were previously identified in European populations, explained 5.1% of circulating vitamin B12 variance (30). Six SNPs were selected as instrumental variables for vitamin D, explaining 7.5% of the variance of its concentration (31). The detailed information for genetic instrumental variables used in this study was presented in Table 1.

**Table 1:** Vitamins- and minerals-associated SNPs used as genetic instruments in the Mendelian randomization analyses.

**SNP**	**Chr**	**Pos**	**Effect allele**	**Other allele**	**EAF**	**Beta**	**SE**	***P***
**Vitamins**				
**Vitamin A** **(****28****)**				
rs10882272	10	95348182	C	T	0.35	−0.03	0.004	7.80E-12
rs1667255	18	29187279	C	A	0.31	0.03	0.004	6.35E-14
**Vitamin B12** **(****30****)**				
rs2270655	4	146576418	G	C	0.941	0.066	0.016	3.50E-05
rs1141321	6	49412433	C	T	0.627	0.061	0.007	1.40E-16
rs1801222	10	17156151	G	A	0.593	0.11	0.007	1.10E-52
rs34324219	11	59623378	C	A	0.881	0.21	0.012	8.80E-71
rs12272669	11	71392610	A	G	0.002	0.51	0.086	3.00E-09
rs41281112	13	100518634	C	T	0.948	0.17	0.016	9.60E-27
rs3742801	14	74759006	T	C	0.294	0.045	0.008	5.30E-08
rs2336573	19	8367709	T	C	0.031	0.32	0.021	1.10E-51
rs602662	19	49206985	A	G	0.596	0.16	0.008	4.10E-96
rs1131603	22	31018975	C	T	0.055	0.19	0.017	4.30E-28
**Vitamin D** **(****31****)**				
rs3755967	4	72828262	T	C	0.28	−0.089	0.002	4.74E-343
rs12785878	11	70845097	T	G	0.75	0.036	0.002	3.80E-62
rs10741657	11	14871454	A	G	0.4	0.031	0.002	2.05E-46
rs17216707	20	52165769	T	C	0.79	0.026	0.003	8.14E-23
rs10745742	12	94882660	T	C	0.4	0.017	0.002	1.88E-14
rs8018720	14	38625936	C	G	0.82	−0.017	0.003	4.72E-09
**Vitamin E** **(****29****)**				
rs602662	19	49206985	G	C	0.15	0.04	0.01	7.80E-12
rs1131603	22	31018975	T	C	0.21	0.03	0.01	1.40E-10
**Minerals**				
**Calcium** **(****25****)**				
rs780094	2	27741237	T	C	0.42	0.017	0.003	1.0E-10
rs1550532	2	234264848	C	G	0.31	0.018	0.003	8.0E-11
rs1801725	3	122003757	T	G	0.15	0.071	0.004	9.0E-86
rs17711722	7	65271197	T	C	0.47	0.015	0.003	8.0E-9
rs10491003	10	9328651	T	C	0.09	0.027	0.005	5.0E-9
rs7481584	11	3029089	G	A	0.3	0.018	0.003	1.0E-10
rs7336933	13	42559076	G	A	0.15	0.022	0.004	9.0E-10
rs1570669	20	52774427	G	A	0.66	0.018	0.003	9.0E-12
**Magnesium** **(****32****)**				
rs4072037	1	155162067	T	C	0.54	0.01	0.001	2.0E-36
rs448378	3	169100899	A	G	0.53	0.004	0.001	1.3E-08
rs13146355	4	77412140	A	G	0.44	0.005	0.001	6.3E-13
rs11144134	9	77499796	C	T	0.08	0.011	0.001	8.2E-15
rs3925584	11	30760335	T	C	0.55	0.006	0.001	5.2E-16
rs7965584	12	90305779	A	G	0.71	0.007	0.001	1.1E-16
**Zinc** **(****27****)**				
rs1532423	8	86268313	A	G	0.43	0.178	0.026	9.0E-12
rs2120019	15	75334184	T	C	0.81	0.287	0.033	1.5E-18
**Selenium** **(****27****)**				
rs248381	5	78337225	A	G	0.5082	−0.025	0.003	3.01E-13
rs17823744	5	78344976	A	G	0.8719	−0.045	0.005	9.91E-17
rs7700970	5	78411324	T	C	0.2942	0.03	0.004	2.21E-11
**Copper** **(****27****)**				
rs1175550	1	3691528	G	A	0.2264	0.198	0.032	5.03E-10
rs2769264	1	151344741	G	T	0.1851	0.313	0.034	2.63E-20
**Iron** **(****24****)**				
rs1799945	6	26091179	G	C	0.15	0.189	0.01	1.1E-81
rs1800562	6	26093141	A	G	0.067	0.328	0.016	2.72E-97
rs855791	22	37462936	G	A	0.554	0.181	0.007	1.32E-139

*SNPs, single nucleotide polymorphisms; Chr, chromosome; Pos, position; EAF, effect allele frequency; SE, standard error*.

### Ascertainment of Sarcopenia

The individual-level phenotypic data on sarcopenia along with its related traits (i.e., grip strength and appendicular lean mass), the relevant confounding factors (i.e., age, sex and relatedness), and the genotypic data are all available in the UK Biobank website (http://www.nealelab.is/uk-biobank). Sarcopenia was defined by referencing to the 2019 definition given by the European Working Group on Sarcopenia in Older People (EWGSOP) (33) using the phenotypic data from the UK Biobank. Participants were diagnosed with confirmed sarcopenia if they had both low grip strength and low muscle mass. Low grip strength was defined as a maximum handgrip strength <27 kg in male and <16 kg in female patients. Skeletal muscle mass index (SMI) was derived from appendicular lean mass (kg) divided by height (m) squared. Low muscle mass was defined as an SMI <7.0 kg/m^2^ in men and <5.5 kg/m^2^ in women. Participants who had non-white European ancestry (to minimize confounding by ancestry), or sex mismatch, or excess heterozygosity, or data deficiency, or second-degree relatives (22, 23) were excluded.

### Statistical Analysis

#### Outcome Data Analysis

The relations between genetically determined levels of circulating micronutrients (calcium, magnesium, selenium, copper, iron, zinc, Vitamin A, Vitamin B12, Vitamin D and Vitamin E) and sarcopenia were examined by logistic regression with adjustments for age, sex, genotype measurement batch, and 20 genetic principal components. In order to explicitly account for the possible population stratification, the genetic principal components that were pre-calculated based on selected genome-wide genotype markers were included.

#### Two-Sample MR Analysis

The two-sample MR analysis was carried out to estimate the potential causal associations between the concentrations of serum micronutrients and the risk of sarcopenia. The principal analyses were conducted by the inverse-variance weighted (IVW) approach, which is to estimate the effect of each SNP on the outcome by computing the Wald ratio and to perform a meta-analysis for the combined causal effect using the inverse variance of SNPs as weights (34). In IVW analysis, the random-effects model, which provides the most precise and unbiased estimates, was employed. The random-effects model assumes that all SNPs are valid instrumental variables (35) or the horizontal pleiotropy is balanced (36).

#### Sensitivity MR Analyses for Pleiotropy

In addition to the two-sample MR analysis, other MR methods were also employed as sensitivity analysis to strengthen the causal inference, each relying on different assumptions to provide confidence in result robustness. In sensitivity analyses, the weighted median method was used first as a proof for IVW analysis, which calculates the combined causal effect of exposure on the outcome based on the weighted median value of SNP-specific estimates, as long as >50% of the selected SNPs are valid variants (37). Furthermore, the MR Egger analysis was carried out to identify both directional and horizontal pleiotropy. Since the MR analysis is based on the hypothesis that the associations between genetic variants and the outcome are due to the pathway through the exposure, any other pathway in the relationship may result in a bias for the estimates in which the pleiotropy occurs. The MR-Egger method is to test the pleiotropic effects by investigating whether the intercept of the association between exposure and outcome is different from zero (38). The leave-one-out sensitivity analysis was carried out by eliminating a single variant from the analysis one by one, and the fluctuation of estimates in response to the elimination of each variant implies the possibility of outlier variant in the causal estimation. It is noteworthy that five exposures (vitamin A, vitamin E, zinc, selenium and copper) with <3 SNPs in their instrumental variables were excluded from the sensitivity analysis.

#### Reported Results and Software

The odds ratio (OR) with 95% confidence interval (CI) per standard deviation (SD) increase in the exposures is presented as the outcome. *P*-value <0.05 was considered statistically significant. All statistical tests were two-tailed. The two-sample MR analyses were carried out using the Mendelian Randomization package in R (version 3.6.3) (39). The associations between genetic variants and sarcopenia were established via the PLINK (version 2.0) software.

## Results

### Participants

Among 487,413 UK Biobank participants, 11,379 participants (2.3%) failed to provide information on grip strength, standing height or fat-free mass. After exclusion criteria were applied, a total of 378,635 individuals of European descent were included (mean age: 56.89 years; male: 46.2%). Of them 577 (0.2%) had a diagnosis of sarcopenia. The flowchart of participants selection is shown in Supplementary Figure 1. The characteristics of the included individuals are presented in Table 2.

**Table 2:** Participant characteristics at recruitment.

**Characteristics**	
All subjects at recruitment, *n*	378,635
Age, mean (SD), years	56.89 ± 7.99
BMI, mean (SD), kg/m^2^	27.41 ± 4.74
Female sex, no. (%)	203,296 (53.8%)
Sarcopenia, no. (%)	577 (0.2%)

*SD, standard deviation; BMI, body mass index*.

### Causal Effects of the Levels of Serum Micronutrients on Sarcopenia

**Figure 1:**
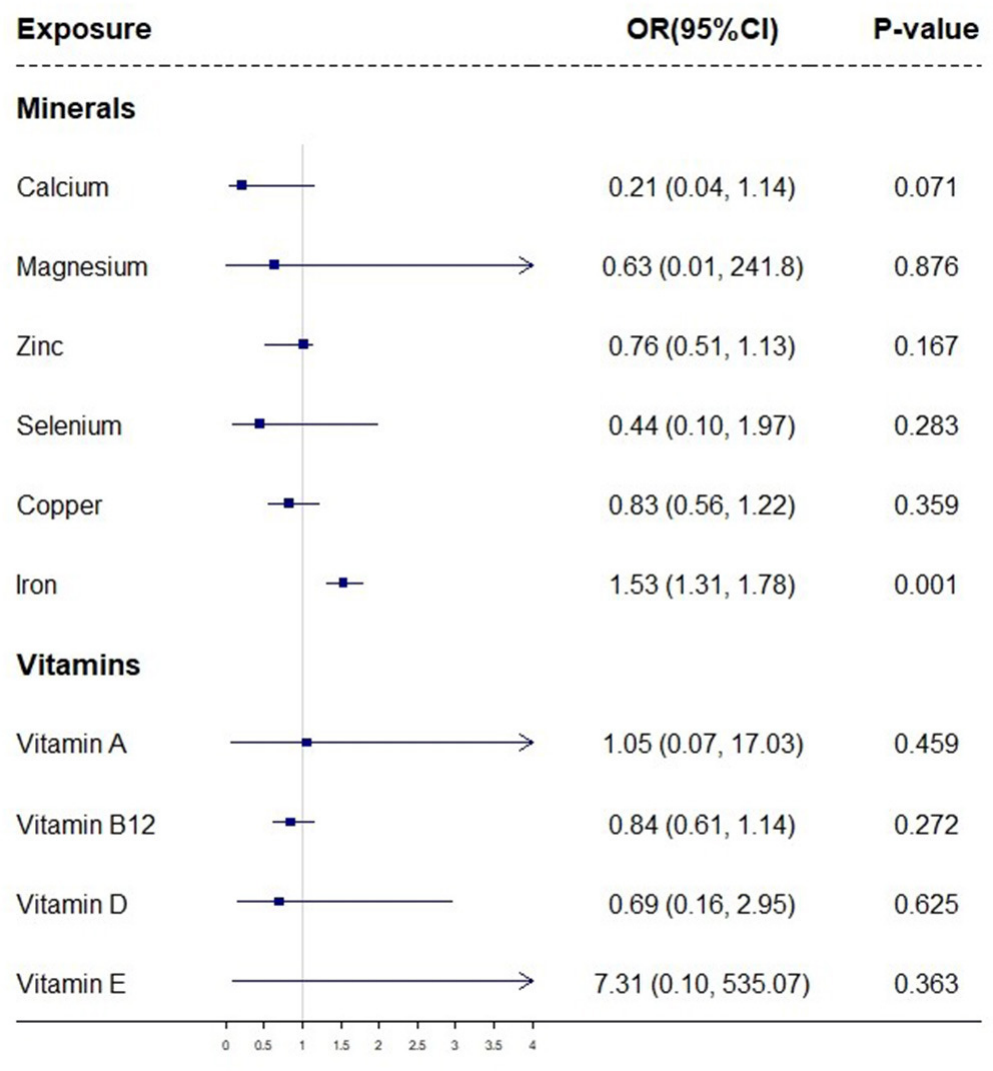
A forest plot showing associations between genetically determined levels of micronutrients and sarcopenia based on IVW MR analysis. The odds ratios (ORs) with their 95% confidence intervals (CIs) are scaled to 1-standard deviation (SD) increase in blood micronutrient level.

Among the micronutrients examined, a genetically predicted higher iron level was found to be positively associated with sarcopenia in IVW analysis (Figure 1; Supplementary Table 1). The ORs of sarcopenia was 1.53 (95% CI, 1.31–1.78; *P* = 0.001) per SD increase in the serum iron level. The serum calcium level showed a potential inverse relationship with sarcopenia (OR = 0.21 per one SD increase of serum calcium level, 95% CI: 0.04–1.14, *P* = 0.071), although not reaching the significance level yet. The genetic predisposition to high circulating levels of other remaining serum micronutrients (magnesium, selenium, copper, zinc, Vitamin A, Vitamin B12, Vitamin D, and Vitamin E) showed no significant associations with sarcopenia (Figure 1).

### Sensitivity Analysis

As shown in Supplementary Table 1, the MR Egger analysis reached similar results on the causal associations between serum iron levels and sarcopenia (OR = 1.57; 95% CI, 1.01–2.44; *P* = 0.046). The weighted median (OR = 2.21) estimates on the association between serum iron and sarcopenia were consistent with IVW and MR Egger analyses, albeit not statistically significant.

The leave-one-out analysis was carried out by eliminating a single genetic variant at a time, in order to identify the SNP that is primarily affected by pleiotropy. The results of IVW in the leave-one-out analysis showed that the *P*-values of intercepts in the associations between serum iron levels and sarcopenia were markedly significant when rs1799945, rs1800562 and rS855791 were removed separately (Supplementary Figure 2).

## Discussion

Our MR analysis on the ten serum micronutrients suggested a positive association between the genetically elevated concentration of circulating iron and the risk of sarcopenia, which was robust under different sensitivity analyses.

### Comparison With Previous Studies

The literature on the association between the circulating iron level and sarcopenia is scarce and inconclusive. A longitudinal study covering 698 elderly participants from the InCHIANTI study rejected any association between the baseline serum iron level and degradation of physical function based on a follow-up of 3 months (40). On the contrary, recent clinical studies indicated a significant relationship between iron accumulation and grip strength and sarcopenia. A study in 2014 covering 1,380 middle-aged and elderly Korean female participants reported that sarcopenia was significantly associated with an increased serum ferritin level, and the risk of individuals with elevated serum ferritin (serum ferritin is a commonly-used clinical indicator for evaluating the iron load of human being) was 2.02-fold of those with a normal serum ferritin level (41). In 2016, a study covering 300 individuals who received hemodialysis found that the forearm grip strength had a significantly negative association with the serum ferritin level (42). In 2017, a study including 639 Italian elderly participants (>65 years) reported that sarcopenia patients had a significantly increased serum ferritin level (43). The inconsistency among different studies may be explained by the variations in study design and outcome measures. In our study, MR analysis was employed to avoid biases that often occur in observational studies and the utilization of data from large-scale genetic consortia. However, our results did not imply any causal effect of other remaining serum micronutrients on the risk of sarcopenia.

### Possible Explanations

Several explanations may account for the association between the genetically predicted serum iron level and the risk of sarcopenia. The skeletal muscle atrophy or injury caused by iron accumulation is likely to be correlated with mitochondrial function, oxidative damage, and the ubiquitin proteasome pathway (44–47). The mitochondria in skeletal muscle cells can not only supply energy for muscle contraction but also regulate catabolic pathways and balance the intracellular oxidative reaction. Under physiological conditions, the non-heme iron that supports the synthesis of mitochondrial coenzymes is mainly provided by mitochondria. However, iron accumulation increases with age, and mitochondria as the main organelles for iron uptake are more likely to develop iron accumulation (48). It is believed that iron excess can generate massive reactive oxygen species (ROS) by Fenton reaction, which can further lead to RNA and DNA damage, and changes of protein conformation and lipid peroxidation. As a consequence, it will result in cell damage and even apoptosis. As suggested by several earlier studies, oxygen-free radicals produced by iron accumulation can cause mitochondrial RNA peroxidation and further induce the opening of mitochondrial permeability transition pores (mPTP), which may lead to the release of cytochrome C into the cytoplasm, the activation of caspase-3, and eventually the apoptosis of skeletal muscle cells (49–52). In 2016, Ikeda et al. reported a decrease of skeletal muscle mass in mouse model resulted from iron accumulation, which could be attributed to the molecular mechanism of the induction of oxidative stress and inhibition of the Akt-FOXO3a pathway (i.e., up-regulation of atrogin-1 and MuRF1). Silencing of FOXO3a expression in C2C12 myotube cells or application of ROS scavenger can inhibit the iron-induced expression of 200 atrogin-1 and MuRF1, as well as prevent cell atrophy (53). It has been demonstrated that the consumption of iron-enriched food and the impaired tissue iron metabolism can both promote high iron stores (54). To sum up, the effect of iron accumulation on sarcopenia is multifactorial, and in our study, the effect of an elevated serum iron level in increasing the risk of sarcopenia was observed.

### Strengths and Limitations

Firstly, the primary strength of this study is attributed to the application of the MR method. From our two-sample MR analysis, we derived data from multiple large-scale GWAS datasets and thus improved the precision of SNPs selection and the power of statistical analysis. In comparison with conventional observational studies, the MR method is more resistant to biases caused by confounding factors and reverse causation. Secondly, we examined the linkage disequilibrium of the included genetic variants. Thirdly, the weighted median analysis was carried out as proof for the observed causalities in IVW analysis and the MR-Egger and leave-one-out approaches were employed to control the bias caused by pleiotropic effects in sensitivity analyses. Finally, the bias of population stratification was minimized in our study as our sample was restricted to participants of European descent in the UK Biobank.

Nevertheless, limitations of this study should also be highlighted. First of all, despite the use of multiple MR approaches to prevent confounding from pleiotropy, the residual bias cannot be fully eliminated, as it is an established limitation of the MR method (55). For example, the micronutrients-related SNPs affect sarcopenia-related outcomes through other causal pathways than through micronutrients exposure cannot be entirely ruled out. Secondly, the association between the serum iron level and sarcopenia observed in IVW analysis was not statistically significant in weighted median analysis. This might be attributed to the assumption of IVW that all the SNPs satisfy the genetic instrumental assumptions in MR analysis, while the weighted median method adopts the hypothesis that over half of the SNPs are valid. Finally, due to the low initial response rate and a degree of healthy responder bias in the UK biobank study, the prevalence of sarcopenia was relatively low in our analysis (56), so was the proportion of variance in the micronutrients explained by genetic variants (ranging from 8% for zinc to 0.37% for calcium). Although no causal association between the levels of nine serum micronutrients (calcium, magnesium, selenium, copper, zinc, Vitamin A, Vitamin B12, Vitamin D, and Vitamin E) and sarcopenia was observed, we could not completely rule out the possibility that our study may not have adequate power to detect a weak association.

### Clinical and Research Implications

Our findings implied a positive association between the genetically predicted iron level and the risk of sarcopenia, which supports a logical speculation that down-regulating serum iron may be beneficial in reducing the risk of sarcopenia. The decrease in iron overload by caloric restriction points out an important direction for sarcopenia intervention (47, 57). However, a recent animal study found that the oral administration of an iron chelator (deferiprone) failed to modulate iron in muscles of old mice model, which indicated the complexity of restricting iron under the conditions of muscle aging and regeneration. Therefore, more comprehensive researches are expected in the future to confirm our findings and elucidate the underlying biological mechanisms, so that appropriate monitoring, treatment and prevention strategies can be developed to effectively manage and control the burden of sarcopenia patients.

## Conclusion

This is the first comprehensive two-sample MR study that investigated the potential associations between genetically predicted concentrations of ten micronutrients and the risk of sarcopenia. From our comprehensive analyses, an increased risk of sarcopenia was observed in relation to a genetically higher concentration of iron, which was robust under different sensitivity analyses. However, further research is warranted to confirm our findings and elucidate the underlying mechanisms.

## Data Availability Statement

The original contributions presented in the study are included in the article/Supplementary Materials, further inquiries can be directed to the corresponding author/s. All relevant GWAS summary statistic data are included in Table 1. The data underlying the results for the UK Biobank presented in the study are available from the UK Biobank: https://www.ukbiobank.ac.uk/, for researchers who meet the criteria for access to the data.

## Ethics Statement

The studies involving human participants were reviewed and approved by North West Center for Research Ethics Committee (11/NW/0382). The patients/participants provided their written informed consent to participate in this study.

## Author Contributions

HL: concept and design. TS, WL, HH, JW, YW, and HL: acquisition, analysis, or interpretation of data, and critical revision of the manuscript for important intellectual content. WL, TS, and HL: drafting of the manuscript. TS: statistical analysis. All authors have revised the article critically for important intellectual content and approved the final version to be published.

## Funding

This work was supported by the National Natural Science Foundation of China (81930071, 81902265, 82072502, and U21A20352), the Key Research and Development Program of Hunan Province (2021SK2017), and the Youth Science Foundation of Xiangya Hospital (2021Q14). The funding source had no role in the design and conduct of the study; collection, management, analysis, and interpretation of the data; praparation, review, or approval of the manuscript; and the decision to submit the manuscript for publication.

## Author Disclaimer

The interpretation of these data is the sole responsibility of the authors.

## Conflict of Interest

The authors declare that the research was conducted in the absence of any commercial or financial relationships that could be construed as a potential conflict of interest.

## Publisher's Note

All claims expressed in this article are solely those of the authors and do not necessarily represent those of their affiliated organizations, or those of the publisher, the editors and the reviewers. Any product that may be evaluated in this article, or claim that may be made by its manufacturer, is not guaranteed or endorsed by the publisher.
