# Supplementary material for: Causal Relationship of Genetically Predicted Serum Micronutrients Levels With Sarcopenia: A Mendelian Randomization Study
Source: Front Nutr. 2022 Jun 22;9:913155. doi: 10.3389/fnut.2022.913155 (PMC9257254; doi:10.3389/fnut.2022.913155)
Supplement: Supplementary file 1 [file Data_Sheet_1.docx]

Supplementary Material

**Supplementary Table 1.** Two-sample Mendelian randomization estimations showing the effect of Nutrients on sarcopenia. 2

**Supplementary Table 2.** Sample size of genetic instruments for serum micronutrient in the two-sample MR analysis. 3

**Supplementary Figure 1.** Flowchart of participants selection. 4

**Supplementary Figure 2.** Mendelian randomization leave-one-out sensitivity analysis for the effect of serum iron levels on sarcopenia. 5

**Supplementary Figure 3.** Mendelian randomization analysis for the effect of serum iron levels on sarcopenia. 6

**Supplementary Table 1.** Two-sample Mendelian randomization estimations showing the effect of Nutrients on sarcopenia

| **Exposure** | **IVW** | | **Weighted median** | | **MR Egger** | |
| --- | --- | --- | --- | --- | --- | --- |
|  | **OR (95% CI)** | **P** | **OR (95% CI)** | **P** | **OR (95% CI)** | **P** |
| **Vitamins** |  |  |  |  |  |  |
| Vitamin A (2 SNPs) | 1.05 (0.07 to 17.03) | 0.459 | - | - | - | - |
| Vitamin B12 (10 SNPs) | 0.84 (0.61 to 1.14) | 0.272 | 0.79 (0.53 to 1.19) | 0.265 | 0.83 (0.51 to 1.37) | 0.497 |
| Vitamin D (6 SNPs) | 0.69 (0.16 to 2.95) | 0.625 | 0.71 (0.18 to 2.80) | 0.623 | 0.39 (0.02 to 6.27) | 0.543 |
| Vitamin E (2 SNPs) | 7.31 (0.10 to 535.07) | 0.363 | - | - | - | - |
| **Minerals** |  |  |  |  |  |  |
| Calcium (8 SNPs) | 0.21 (0.04 to 1.14) | 0.071 | 0.30 (0.03 to 2.95) | 0.301 | 0.31 (0.01 to 9.78) | 0.537 |
| Magnesium (6 SNPs) | 0.63 (0.01 to 241.8) | 0.876 | 0.96 (0.01 to 12662.5) | 0.967 | 0.01 (0.00 to 1.81E+5) | 0.514 |
| Zinc (2 SNPs) | 0.76 (0.51 to 1.13) | 0.167 | - | - | - | - |
| Selenium (3 SNPs) | 0.44 (0.10 to 1.97) | 0.283 | 0.53 (0.11 to 2.55) | 0.429 | 1.09 (0.04 to 26.70) | 0.963 |
| Copper (2 SNPs) | 0.83 (0.56 to 1.22) | 0.359 | - | - | - | - |
| **Iron (3 SNPs)** | 1.53 (1.31 to 1.78) | 0.001 | 2.21 (0.40 to 12.39) | 0.185 | 1.57 (1.01 to 2.44) | 0.046 |

CI: confidence intervals; IVW: inverse variance-weighted method; OR: odds ratio.

| **Micronutrients** | **Sample size** |
| --- | --- |
| Vitamin A | 5,006 |
| Vitamin B12 | 82,917 |
| Vitamin D | 79,366 |
| Vitamin E | 7,781 |
| Calcium | 39,400 |
| Magnesium | 23,829 |
| Zinc | 2,603 |
| Selenium | 2,603 |
| Copper | 2,603 |
| Iron | 48,972 |

**Supplementary Table 2.** Sample size of genetic instruments for serum micronutrient in the two-sample MR analysis.

MR, Mendelian randomization.


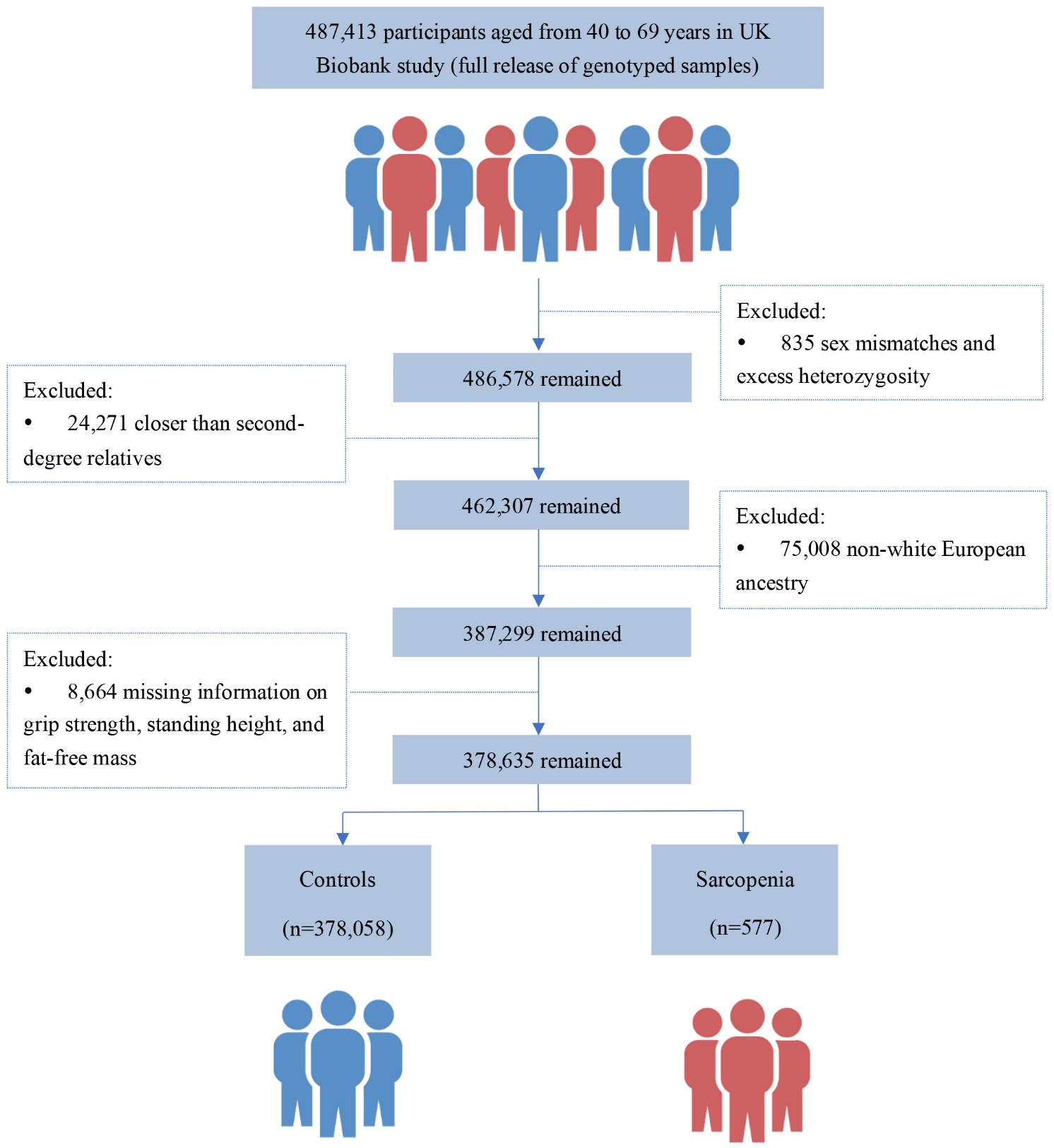


**Supplementary Figure 1.** Flowchart of participants selection.


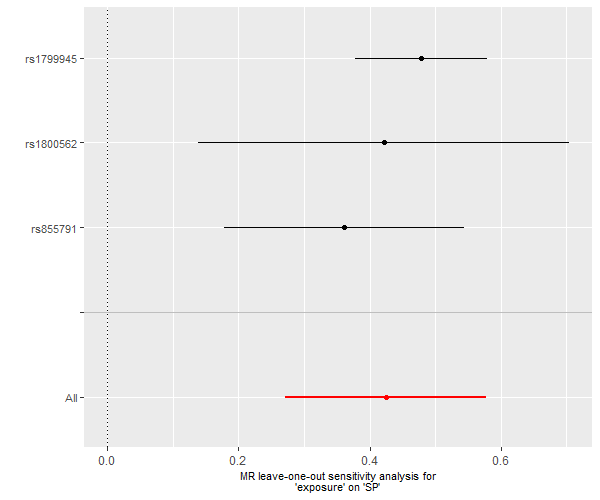


**Supplementary Figure 2.** Mendelian randomization leave-one-out sensitivity analysis for the effect of serum iron levels on sarcopenia.


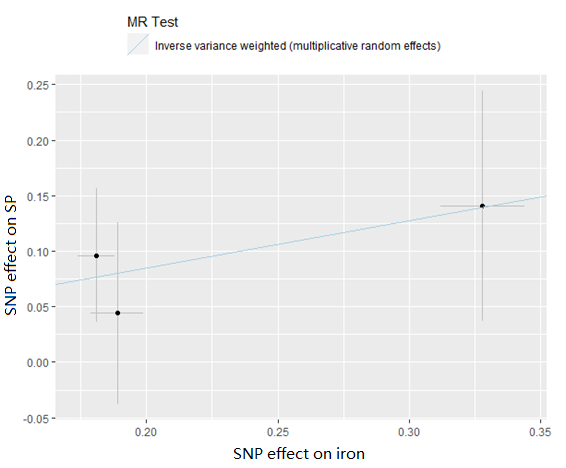


**Supplementary Figure 3.** Mendelian randomization analysis for the effect of serum iron levels on sarcopenia.
